# Diagnosis and treatment of autonomic failure, pain and sleep disturbances in Parkinson’s disease: guideline “Parkinson’s disease” of the German Society of Neurology

**DOI:** 10.1007/s00415-024-12730-5

**Published:** 2025-01-03

**Authors:** Alessandra Fanciulli, Friederike Sixel-Döring, Carsten Buhmann, Florian Krismer, Wiebke Hermann, Christian Winkler, Dirk Woitalla, Wolfgang H. Jost, German Parkinson’s Guideline Group, Mathias Bähr, Jos Becktepe, Daniela Berg, Kathrin Brockmann, Andrés Ceballos-Baumann, Joseph Claßen, Cornelius Deuschl, Günther Deuschl, Richard Dodel, Georg Ebersbach, Beelitz-Heilstätten, Carsten Eggers, Thilo van Eimeren, Bruno Fimm, Ann-Kristin Folkerts, Madeleine Gausepohl, Bad Segeberg, Alkomiet Hasan, Rüdiger Hilker-Roggendorf, Matthias Höllerhage, Franziska Hopfner, Wolfgang Jost, Elke Kalbe, Jan Kassubek, Stephan Klebe, Christine Klein, Martin Klietz, Thomas Köglsperger, Andrea Kühn, Paul Krack, Florian Krismer, Gregor Kuhlenbäumer, Johannes Levin, Inga Liepelt-Scarfone, Paul Lingor, Kai Loewenbrück, Matthias Löhle, Stefan Lorenzl, Sylvia Maaß, Walter Maetzler, Regina Menzel, Philipp T. Meyer, Brit Mollenhauer, Manuela Neumann, Per Odin, Tiago Outeiro, Monika Pötter-Nerger, René Reese, Kathrin Reetz, Olaf Rieß, Viktoria Ruf, Anja Schneider, Christoph Schrader, Alfons Schnitzler, Klaus Seppi, Alexander Storch, Lars Tönges, Thilo van Eimeren, Uwe Walter, Tobias Wächter, Tobias Warnecke, Florian Wegner, Karsten Witt, Dirk Woitalla, Kirsten Zeuner, Claudia Trenkwalder, Günter Höglinger

**Affiliations:** 1 Department of Neurology, Innsbruck Medical University, Anichstrasse 35, 6020 Innsbruck, Austria; 2 Paracelsus-Elena-Klinik, Kassel, Germany; 3 Department of Neurology, Philipps-Universität, Marburg, Germany; 4 Department of Neurology, University Clinic Eppendorf, Hamburg, Germany; 5 Department of Neurology, Rostock University Medical Center, Rostock, Germany; 6 Deutsches Zentrum für Neurodegenerative Erkrankungen (DZNE) Rostock/Greifswald, Rostock, Germany; 7 Department of Neurology, Lindenbrunn Hospital, Coppenbrügge, Germany; 8 Kathol. Kliniken der Ruhrhalbinsel, Essen, Germany; 9 Parkinson-Klinik Ortenau, Wolfach, Germany; 10 Department of Neurosurgery, University Medical Center Göttingen, Göttingen, Germany; 11 Department of Neurology, LMU University Hospital, Ludwig-Maximilians-Universität (LMU) München, Munich, Germany; 12 Deutsches Zentrum für Neurodegenerative Erkrankungen (DZNE), Munich, Germany; 13 Munich Cluster for Systems Neurology (SyNergy), Munich, Germany

**Keywords:** Parkinson’s disease, Orthostatic hypotension, Supine hypertension, Urinary incontinence, Nocturia, Erectile dysfunction, Constipation, Pain, Sleep, Daytime sleepiness, REM-sleep behavior disorder

## Abstract

**Background and objective:**

Non-motor symptoms frequently develop throughout the disease course of Parkinson’s disease (PD), and pose affected individuals at risk of complications, more rapid disease progression and poorer quality of life. Addressing such symptom burden, the 2023 revised “Parkinson’s disease” guideline of the German Society of Neurology aimed at providing evidence-based recommendations for managing PD non-motor symptoms, including autonomic failure, pain and sleep disturbances.

**Methods:**

Key PICO (Patient, Intervention, Comparison, Outcome) questions were formulated by the steering committee and refined by the assigned authors. Recommendations were drafted based on relevant studies, systematic reviews, meta-analyses and high-quality guidelines identified by the literature search. They were subsequently reviewed, revised, and voted by the Guideline Group in online consensus conferences. Consensus was achieved in case of > 75% agreement among the group members. The consensus was considered strong, if agreement was > 95%.

**Results:**

The guideline entails: (i) 10 PICOs and 23 recommendations on the diagnosis and treatment of urogenital, cardiovascular and gastrointestinal autonomic failure; (ii) four PICOs and four recommendations on the possible types of pain in PD individuals, their diagnosis and treatment; (iii) 11 PICOs and 11 recommendations on the screening, diagnosis and treatment of sleep disturbances and excessive daytime sleepiness in PD individuals, as well as on their prognostic implications. Thirty-one out of 38 recommendations achieved a strong consensus.

**Conclusion:**

The current German PD guideline provides a practice-oriented and etiology-driven stepwise approach to the diagnosis and treatment of autonomic failure, pain and sleep disturbances in PD individuals.

## Introduction

Non-motor symptoms, including REM-sleep behavior disorders (RBD), insomnia, autonomic failure, and sometimes pain, may precede the development of Parkinson’s disease (PD) motor symptoms by years and their recognition often contributes to an early identification of affected PD individuals [19, 67]. The frequency and severity of PD non-motor symptoms however typically increases with disease duration, and has been associated with morbidity, mortality, more rapid disease progression and overall poorer quality of life of PD individuals and their caregivers [15].

Lower urinary tract symptoms are a common non-motor symptom in individuals with PD and atypical parkinsonian disorders and are divided into disturbances of the storage phase, mostly due to bladder detrusor hyperactivity, and disturbances of the voiding phase due to detrusor-sphincter dyssynergia or hypocontractile detrusor [1, 88, 167]. Neurogenic bladder dysfunction refers to any disturbance of bladder function due to impaired neural control and needs to be distinguished from non-neurogenic bladder disorders, especially in elderly individuals.

Male erectile dysfunction is defined as the inability to achieve and maintain a penile erection sufficient for a satisfactory sexual intercourse [44]. Erectile dysfunction has a major impact on the well-being of affected individuals and may be accompanied by decreased libido and ejaculation difficulties. The incidence of erectile dysfunction increases exponentially with age [26] due to concomitant vascular and other contributing factors. Up to 80% of male PD individuals have been indeed found to suffer from erectile dysfunction [191], highlighting a major, though often unspoken, therapeutic need in these individuals.

Neurogenic orthostatic hypotension (OH) is another non-motor feature affecting one-third of PD individuals [166, 241]. In most cases, OH manifests with non-specific symptoms and complaints, such as orthostatic dizziness, light-headedness or blurred vision, but it may cause orthostatic syncope, increasing risk of injurious falls and their complications, often leading to hospitalization. Neurogenic OH in PD develops after a degeneration of the post-ganglionic sympathetic neurons constituting the efferent branch of the baroreflex arch, but concomitant non-neurogenic factors including infections, dehydration and cardiac diseases, may unmask or worsen OH, especially in elderly individuals [112]. Parkinsonian medications and other frequently used CNS-active drugs (i.e. hypnotics, antidepressants, neuroleptics, opioids) may also aggravate orthostatic intolerance [187]. Due to progressive baroreflex dysfunction and other, to date not fully understood, additional mechanisms, about 50% of individuals with neurogenic OH paradoxically develop hypertensive blood pressure (BP) levels in the supine position [59, 66] that can be severe and last for several hours during sleep (nocturnal hypertension). Drugs to treat OH can unmask or aggravate supine hypertension, but this often develops independently of any anti-hypotensive treatment [62]. Possible complications of supine and nocturnal hypertension include hypertensive emergencies, chronic end-organ damage and increased pressure natriuresis overnight, this in turn aggravating nocturia and early morning OH symptoms—a phenomenon that often remains unrecognized by affected individuals and their caregivers.

Constipation is the most frequent autonomic feature affecting PD individuals and is determined by both neurogenic and non-neurogenic factors, such as reduced mobility and abdominal muscle tone, a diet poor in fibers and insufficient fluid intake, all contributing to a prolonged gastrointestinal transit time and sometimes megacolon [251]. Antiparkinsonian medications, especially anticholinergics [102, 116], as well as several other frequently prescribed medications, such as opioids, tricyclic antidepressants, calcium-containing antacids, antihypertensives, spasmolytics, sympathomimetics, and diuretics, may also worsen constipation. Anismus is defined by the lack of relaxation or involuntary contraction of the anal sphincter during defecation and may rarely cause an ‘outlet constipation’ in PD individuals [250].

Pain is another common non-motor symptom in PD. Different pain phenotypes have been described in PD individuals with complex and often multifactorial etiologies. Some painful conditions, such as arthrosis of the spine or joints, may be aggravated by PD-related motor impairment and may further limit the mobility of affected individuals in everyday life. A bidirectional influence in fact exists between PD-related and PD-unrelated pain determinants [29], ultimately preventing an exact etiological pain classification.

Sleep disturbances develop in up to 90% of PD individuals, significantly impacting on their quality of life [15, 17, 118, 196, 264], caregivers’ burden [25, 202, 220, 266] and disease progression, especially in case of RBD [157, 176]. Daytime sleepiness, due to sleep impairment, medications or as a separate entity caused by PD itself, further restricts social interaction, participation, mobility, professional and leisure activities of PD individuals.

Acknowledging the symptom burden and health hazard derived by PD non-motor symptoms, the 2023 edition of the “Parkinson’s disease” guideline of the German Society of Neurology (Deutsche Gesellschaft für Neurologie, DGN) for the first time entailed recommendations for the diagnosis and treatment of non-motor PD symptoms, including autonomic failure, pain and sleep disturbances.

This article is an abridged translation of the original guideline in German language and focuses on the diagnosis and treatment of autonomic failure, pain and sleep in individuals with PD. The full German version of the guideline is available under www.dgn.org/leitlinien and the AWMF (Arbeitsgemeinschaft wissenschaftlicher Medizinischer Gesellschaften, AWMF version number: 8.0. Level of guideline: S2k. Date of last update: 10/2023. Valid until: 24.10.2028. Edited by: Guidelines Committee of the German Society of Neurology) [93].

### Guideline methodology

During the purposing phase, key PICO (Patient, Intervention, Comparison, Outcome) questions were formulated by the guideline steering committee. The respective chapter authors subsequently refined the PICO wording and performed a comprehensive literature search, identifying pertinent studies, systematic reviews, meta-analyses and high-quality guidelines. The identified literature was further enriched with additional evidence sources provided by the assigned authors. The chapter authors subsequently drafted the background and recommendation texts, which were reviewed, and put to an online vote by all members of the German Parkinson Guideline Group. Recommendations that achieved < 75% consensus required group discussion, revision and re-voting. Recommendations with > 75% agreement among the group members were considered consented. Consensus was “strong", if agreement outreached a 95% quote.

For the purposes of the present work, the dosage ranges of the recommended medications have been added based on the available manufacturer’s information and clinical experience of the authors. Studies assessing the efficacy and safety of different drug schedules in the specific setting of PD were, however, oftentimes missing. Treating physicians are therefore requested to consult the applicable prescribing information in their respective country and to perform a case-by-case risk–benefit assessment when considering prescribing any of the therapeutic options mentioned hereafter.

### The most important recommendations

#### Bladder disturbances

- Non-pharmacological measures to treat neurogenic bladder dysfunction in PD include bladder and pelvic floor training, adjusting fluid intake according to the time of day and avoiding caffeine, alcohol and carbonated drinks.
- Antimuscarinics (preferably solifenacin 5 mg o.i.d, trospium 15–30 mg b.i.d or darifenacin 7.5–15 mg o.i.d., due to their lower risk of cognitive side effects) should be considered for the pharmacological treatment of urinary urge incontinence due to bladder detrusor overactivity in PD.
- β_3_ adrenoceptor agonists (e.g. mirabegron 50 mg o.i.d.) can be considered for treating urinary urge due to bladder detrusor overactivity in PD individuals, who have responded inadequately to antimuscarinics, have not tolerated them or in whom antimuscarinics are contraindicated.
- In patients who have responded inadequately to oral therapy, botulinum toxin A intravesical injection (200 U or customized) may be considered for treating severe urinary urge incontinence, if the individual motor and cognitive performance enables the subsequently likely necessary intermittent catheterization.
- For the non-pharmacological treatment of nocturia, affected individuals should be advised to limit their fluid intake in the second half of the day, avoid alcohol consumption in the evening and sleep in a 10°–20° head-up tilted position.
- For the treatment of nocturnal polyuria, an evening desmopressin administration (5–40 µg o.i.d. nasal spray or 100–800 µg o.i.d. per os) can be considered under close monitoring of blood pressure, serum electrolytes and body weight.
- To treat nocturia due to reduced bladder capacity, treatment with antimuscarinics (preferably solifenacin, trospium or darifenacin due to their lower risk of cognitive side effects) should be considered instead.

#### Orthostatic hypotension

The following stepwise approach should be applied for treating OH in PD:

- Eliminate or treat aggravating factors (e.g., infectious diseases, dehydration).
- Review concomitant medications (if antihypertensives are being used, a dose reduction or discontinuation should be sought).
- Apply non-pharmacological strategies.
- Start anti-hypotensive medications.

#### Constipation

- For treating constipation in individuals with PD, the DGN recommends following the AWMF guideline on ‘Chronic Constipation’.

#### Pain

- The first therapeutic step is an optimization of antiparkinsonian medications.
- Nociceptive pain should be treated according to the 3-step WHO regimen.
- Neuropathic pain should be treated with anticonvulsants and/or antidepressants as indicated in the guidelines for the treatment of neuropathic pain, with preference given to gabapentin 300–1800 mg (rarely up to 3600 mg), especially in case of concomitant restless legs syndrome, and/or duloxetine 60–120 mg, in case of concomitant depression. Gabapentin is preferred over pregabalin due to its proven tolerability in PD and potentially positive influence on motor symptoms [163, 238].
- For severe pain, treatment with prolonged-release oxycodone/naloxone 5/2.5–20/10 mg (rarely up to 40/20 mg) may be considered.
- In the experience of the authors of the present publication, local botulinum toxin application might be considered in single individuals with pain refractory to maximized oral or transdermal treatment, especially in case of on- and off dystonia, muscular pain trigger points, painful myotonus, spasms in the esophagus, pylorus, anal sphincter or hyperactive bladder [29]. In such cases, the botulinum toxin application should be endoscopically guided.

#### Sleep

- Primary sleep disorders such as restless leg syndrome or sleep disordered breathing should be treated according to the corresponding guidelines.
- If motor or non-motor fluctuations determine sleep disturbances, the dopaminergic pharmacotherapy should be adjusted accordingly.
- RBD should be firstly treated by creating a safe sleep environment. Clonazepam (0.125–3 mg) and/or melatonin (2–9 mg) can be considered, bearing in mind possible side effects.
- In case of insomnia or circadian rhythm disorders, underlying causes such as medication side effects and/or primary sleep disorders should be ruled out. If treatable causes have been ruled out, circadian rhythm disorders should be treated with sleep hygiene measures, intensive physical training and light therapy. Starting with the lowest possible dosage, eszopiclone (1 mg), doxepin (25 mg), zolpidem (5 mg), trazodone (50 mg), melatonin (2 mg), venlafaxine (37.5 mg, in case of comorbid depression), nortriptyline (25 mg) or mirtazapine (7.5 mg) can be used for treating insomnia (low level of evidence). Further dose adjustments may be required on an individual basis. Side effects should be carefully monitored at follow-up.

### The guidelines in detail

#### What kind of bladder dysfunction can occur in individuals with PD?

Neurogenic bladder dysfunction is found (depending on the stage of the disease) in around 60% of all individuals with PD [122]. The bladder filling or storage phase is most frequently affected, leading to urinary frequency, nocturia, urinary urge and urge incontinence. Such symptoms impact significantly on the quality of life of affected individuals and often require dedicated, multidisciplinary management. Non-neurogenic bladder disorders (e.g. prostate hyperplasia in men or pelvic floor prolapse in women) must be carefully differentiated from neurogenic bladder disorders in close collaboration with allied specialties such as urology and gynecology. In addition to urological/gynecological consultations, urinalysis is also essential to rule out reversible causes of bladder disturbances such as urinary tract infections, and to screen for proteinuria, diabetes mellitus or space-occupying lesions, in case of persistent hematuria. Keeping a bladder diary for 48–72 h helps to quantify the micturition frequency, urine volume, incontinence episodes, as well as to identify nocturnal polyuria [117], ultimately enabling an accurate estimation of the individual symptomatic burden. Additional examinations include the measurement of post-void residual urine volume by means of bladder ultrasound or in-and-out catheterization, uroflowmetry, and urodynamics. The latter is considered the gold standard in the diagnostic workup of neurogenic bladder disorders.

**Table:** 

Recommendation	
Individuals with PD frequently develop disturbances of the bladder storage phase, including nocturia, urinary frequency, urgency and/or urge incontinence	
Level of consensus: 100%, strong consensus	

#### How to treat bladder dysfunction in PD?

The treatment of bladder dysfunction aims to improve the quality of life of affected individuals by reducing incontinence episodes and to prevent secondary upper urinary tract damage by improving micturition control [167, 193]. Non-pharmacological and behavioral measures such as bladder training, adjusting the amount of fluid intake, avoiding caffeine, alcohol and carbonated drinks can alleviate symptoms associated with neurogenic bladder dysfunction [45, 223, 249]. To avoid social stigmatization due to bladder incontinence, appliances (e.g. condom urinals, pads) can be of help [88]. Antimuscarinics, including oxybutinin, tolterodine, solifenacin, darifenacin and trospium, are the first pharmacological option for treating urinary urgency and urge incontinence due to bladder detrusor overactivity. Possible side effects of antimuscarinics include: increased post-void residual urine volume, xerostomia, constipation and blurred vision [255]. Delirium, confusion and worsening of pre-existing cognitive impairment are further side effects due to the central anticholinergic effect and limit the use of anticholinergics in PD individuals with dementia. Trospium (15–30 mg b.i.d.), which does not cross the blood–brain barrier, as well as solifenacin (5 mg o.i.d), and darifenacin (7.5–15 mg o.i.d.), that selectively stimulate peripheral M3 muscarinic receptors, are less likely to cause cognitive side effects and may be therefore preferred [114, 232]. The post-void residual urine volume should be monitored during the titration phase and after dose changes of antimuscarinics, as this may increase due to drug-induced bladder detrusor weakness [4].

Mirabegron, a β_3_ adrenoceptor agonist administered 50 mg o.i.d., was found to reduce urinary urge incontinence due to detrusor overactivity in different neurological disorders, including PD [43, 150], with an acceptable safety profile [42]. Compared to antimuscarinic drugs, mirabegron was less frequently associated with urinary retention, constipation, cognitive impairment and dry mouth and may be therefore considered as an alternative for patients in whom antimuscarinics are contraindicated, not tolerated [81, 115, 223] or did not sufficiently improve incontinence symptoms [55].

In refractory cases, more invasive options may also be considered. Intravesical botulinum toxin A injection (200 U, or customized) reduces incontinence episodes due to detrusor overactivity [83], but impairs voluntary voiding and leads to urinary retention. Such procedure is therefore only indicated in patients with sufficient hand-dexterity to perform clean intermittent self-catheterizations and not in patients with severe cognitive or motor disorders, or without available caregiver [47, 200]. Percutaneous [33] and transcutaneous [7, 171] tibial nerve stimulation can also improve symptoms of overactive bladder in PD.

In patients reporting frequent nocturnal toilet visits, a bladder diary should be used to differentiate between nocturnal polyuria (i.e. more than 1/3 of the 24-h total urine volume is excreted overnight, e.g. in patients with nocturnal hypertension), a generally increased urine output (> 40 ml/kg body weight, e.g. in uncontrolled diabetes) and a reduced bladder capacity (e.g. in overactive bladder). A combination of more than one cause is common and additional factors that may contribute to nocturia should also be considered as part of the initial assessment. These include nocturnal hypertension, which is found in approximately 50% of PD individuals with OH, evening use of diuretics, sleep apnoea, psychogenic polydipsia and insufficient secretion of antidiuretic hormone (either idiopathic, drug-induced or due to pituitary lesions).

In case of nocturia, patients should be advised to limit their fluid intake in the late afternoon/evening, avoid any evening alcohol consumption and sleep in a 10°–20° head-up tilted position [205]. Bedtime desmopressin (5–40 µg o.i.d. nasal spray or 100–800 µg o.i.d. per os) can be considered in patients with nocturnal polyuria under close BP, serum electrolytes and body weight monitoring [192]. Bedtime antimuscarinics (i.e., trospium 15–30 mg) can be considered in patients with nocturia due to reduced bladder capacity.

**Table:** 

Recommendation	
Non-pharmacological measures for treating neurogenic bladder dysfunction in PD include bladder training, adjusting fluid intake to the daily routine, as well as avoiding caffeine, alcohol and carbonated drinks	
To avoid social stigmatisation due to bladder incontinence, continence appliances should be recommended	
Antimuscarinics (preferably solifenacin, trospium or darifenacin, due to their lower risk of cognitive side effects) should be considered for the pharmacological treatment of urinary urge incontinence due to bladder detrusor overactivity in PD	
β_3_ adrenoceptor agonists (e.g. mirabegron) can be considered for the treatment of urinary urge and urge incontinence due to bladder detrusor overactivity in those PD individuals who have responded inadequately or have not tolerated antimuscarinics, or for whom antimuscarinics are contraindicated	
Intravesical injection of botulinum toxin A can be considered for the treatment of detrusor overactivity in individuals, who have responded inadequately to oral therapy, provided that the patient's motor and cognitive performance enables intermittent self-catheterizations that are likely required after the procedure	
PD individuals with nocturia should be advised to limit fluid intake in the late afternoon/evening, avoid alcohol consumption in the evening, and sleep in a 10°–20° head-up tilted position	
To treat nocturnal polyuria, bedtime desmopressin can be considered under close BP, serum electrolytes and body weight monitoring	
To treat nocturia due to reduced bladder capacity, bedtime antimuscarinics should be considered	
Level of consensus: 100%, strong consensus	

#### How to diagnose erectile dysfunction in PD?

The diagnosis of erectile dysfunction is primarily in the hands of urologists. A detailed medical history is essential to detect modifiable causes and influencing factors (e.g. concomitant SSRIs or β-blockers treatment). A neurological and urological examination should be performed, and signs of peripheral arterial disease assessed. A laboratory examination should include cardiovascular risk factors (fasting glucose, HbA1c, lipid status), common causes of polyneuropathy (renal and liver function parameters, serum protein electrophoresis, vitamin B1, B6, B12 status) and, in patients with gynaecomastia, testosterone levels. If a vascular origin is suspected, a provocation test with intracavernous application of alprostadil (± vascular sonography) may be necessary. Additional electrophysiological examinations (e.g. pudendal nerve SSEP) can help to confirm a neurogenic cause of erectile dysfunction [12, 87].

**Table:** 

Recommendation	
A multidisciplinary approach with colleagues from the urology service is necessary in PD individuals complaining of erectile dysfunction. The basic diagnostic work-up includes a detailed medical history, clinical neurological and urological examination, as well as laboratory testing. Additional electrophysiological examinations may be necessary in single cases	
Level of consensus: 100%, strong consensus	

#### How to treat male erectile dysfunction in PD?

The efficacy of oral phophodiesterase 5 (PDE-5) inhibitors (sildenafil, tadalafil, vardenafil), and in particular of sildenafil (sildenafil 50–100 mg on demand), for treating erectile dysfunction in male individuals PD was sufficiently proven [127, 204] [20, 96, 180, 267]. Indirect comparisons between different PDE-5 inhibitors also suggest that they are equally effective [148]. The occurrence of acute cardiovascular events during intercourse and worsening of OH must however be considered. For this reason, PDE-5 inhibitors with long half-life (tadalafil) should be used with caution in patients with proven OH, as there is a risk of long-lasting arterial hypotension. Intraurethral or intracavernous application of vasodilatory prostaglandins (alprostadil 10–20 µg intra-cavernous injection on demand) may be considered, although there have been no studies in PD individuals and there is an increased risk of priapism and bleeding [12, 149]. Case series and a multicentre double-blind study suggest a positive therapeutic effect of apomorphine (sublingual or subcutaneous) on erectile dysfunction [54, 160]. Penile prosthesis can be considered in single cases [12].

**Table:** 

Recommendation	
Treatment with PDE-5 inhibitors should be considered for the pharmacological treatment of erectile dysfunction in individuals with PD with a low cardiovascular risk during sexual intercourse. A possible unmasking/aggravation of OH should be considered	
Level of consensus: 100%, strong consensus	

#### How to diagnose OH in PD?

OH is defined by a fall in systolic BP ≥ 20 mmHg or diastolic ≥ 10 mmHg within 3 min of head-up tilt test or active standing [77]. Sustained systolic BP values < 90 mmHg upon standing are also considered suggestive of OH [27]. In the expert consensus, two methods are approved for OH detection and should be considered for its diagnosis in PD: the active standing test (also known as *Schellong test*, with BP and heart rate measurements after at least 5 min in the supine position and at least after 1 and 3 min after changing to the upright position [58]) and the head-up tilt test examination (with tilting by at least 60°) [229]. Neurogenic OH is characterized by a very modest or completely abolished heart rate increase despite severe orthostatic BP fall, indicating baroreflex dysfunction [112]. This is identified by a neurogenic OH ratio < 0.5, which is calculated by dividing the supine-to-upright change in heart rate per the supine-to-upright fall in systolic BP [63, 158].

**Table:** 

Recommendation	
In individuals with PD, a Schellong test should be performed to diagnose OH. A tilt table examination can be considered if available, as it provides more comprehensive and precise information	
Level of consensus: 100%, strong consensus	

#### How to treat OH in PD?

OH in PD should be treated with a stepwise approach [27, 57, 254]. First, exacerbating factors such as concomitant infections, anemia, dehydration, and the combination of multiple drugs with BP lowering effect should be excluded or treated.

In a second step, non-pharmacological therapeutic measures should be considered. Rapid drinking of cold water has a significant, though transient, pressor effect lasting about 1 h [101, 140, 203, 206, 263]. Studies showed that salt supplements had no additive effect with respect to increased water intake [125, 181, 265], but no dietary salt restriction should be recommended either. Large meals can produce significant falls in BP (e.g. post-prandial hypotension) [179], and it is therefore advisable to divide the daily caloric intake into several smaller meals. Sleeping in a 10°–20° head up tilted position can also improve orthostatic tolerance the day after [228, 239]. A steeper head-up tilt sleeping position is likely more effective and can be pursued, if well tolerated. Physical counterpressure maneuvers (squatting or crossing the legs and tensing the leg, buttock, abdominal and arm muscles) can be recommended to stabilize the BP in case of dizziness [212, 227, 236, 240]. Wearing an elastic abdominal binder is an additional non-pharmacological option to increase orthostatic BP levels, and more effective than compression stockings [61, 71, 156, 169, 212, 236].

Midodrine (2.5–10 mg b.i.d./t.i.d) is a vascular adrenoceptor agonist and its efficacy to treat OH has been demonstrated in several randomized control trials [75, 98, 110, 129, 183, 256], with safety concerns regarding the exacerbation of supine hypertension and urinary retention. Paresthesia, pruritus, goose bumps and shivering are relatively common, and sometimes unpleasant side effects of midodrine to monitor for.

Fludrocortisone (0.1–0.3 µg o.i.d.) is a synthetic mineralocorticoid agent and represents an additional pharmacological option to combat OH, although with a lower level of evidence regarding its efficacy and safety [110, 198], mostly concerning the exacerbation of supine (and nocturnal) hypertension, and electrolyte disturbances.

Randomized controlled trials, case series and long-term observational studies reported on the efficacy of droxidopa (l-Threo-DOPS, 100–300 mg b.i.d. or t.i.d., rarely up to 600 mg t.i.d.), a synthetic noradrenalin precursor, to treat OH in individuals with PD and other primary autonomic [22, 23, 35, 76, 89, 90, 97, 104, 111, 113, 133, 141, 142, 194] disorders. Based on these studies, Droxidopa was approved by the Food and Drug Administration for the treatment of OH, but is not available in Europe. Like other pressor agents, droxidopa may also exacerbate or worsen supine and nocturnal hypertension.

Smaller case series reported on the efficacy of atomoxetine, yohimbine and pyridostigmine to treat OH in different disorders, including PD [57]. However, the clinical use of the latter drugs is often limited to specialized dysautonomia centers.

**Table:** 

Recommendation	
To treat OH in individuals with PD, a stepwise approach should be followed	
1. Eliminate or treat aggravating and triggering factors (infections, dehydration, etc.)	
2. Review the medication schedule (if anti-hypertensive drugs are being used, a dose reduction or discontinuation should be sought)	
3. Apply non-pharmacological therapeutic measures (see recommendations below)	
4. Start pressor agents (see recommendations below)	
Level of consensus: 100%, strong consensus	
The following non-pharmacological therapeutic measures should be recommended for treating OH in PD	
1. Adequate fluid and salt intake, provided there are no contraindications for this (e.g. cardiac, liver, renal failure)	
2. Abstain from large meals or excessive alcohol consumption	
3. Minimize heat exposure	
4. Sleep in a 10°–20° head-up tilt position	
5. Perform counterpressure manoeuvres in case of orthostatic intolerance (e.g. tensing the legs, buttock, abdominal and arm muscles)	
6. Wear an elastic abdominal binder during daytime (more effective than compression stockings)	
Level of consensus: 95%, consensus	
Midodrine should be considered as pharmacological measure to treat OH in PD	
Level of consensus: 100%, strong consensus	
Fludrocortisone can be considered as pharmacological measure to treat OH in PD	
Level of consensus: 100%, strong consensus	
Droxidopa can be considered as pharmacological measure to treat orthostatic hypotension in individuals with PD (off-label use, not licensed in Europe)	
Level of consensus: 95%, consensus	

#### How to diagnose supine and nocturnal hypertension in PD?

PD individuals newly diagnosed with neurogenic OH, should be screened for the presence of supine and nocturnal hypertension, and such screening should be repeated at regular intervals during the disease course, especially if treatment with pressor agents is started, its dosage is increased, or if individuals with known neurogenic OH develop multiple episodes of nocturia or ankle oedema.

Measuring the BP during the supine phase of the Schellong test already provides initial insights into the presence of supine hypertension. In addition, patients should consider keeping blood pressure diaries with measurements at different times of the day [i.e., in the early morning, at midday and before going to bed, each time with measurements in the supine, (sitting) and standing position] to gain insights into circadian and activity-dependent BP fluctuations. In a second step, a 24-h ambulatory BP monitoring can be considered to determine the presence of nocturnal hypertension and to document the absolute BP values reached over the 24-h. To ensure an accurate interpretation of the exam, patients should be instructed to complete an activity diary on the day of the examination, indicating the times of medication intake (especially antihypertensive and pressor agents), meals, physical activities and any phase in the upright position overnight, e.g. to use the bathroom.

The diagnosis of supine hypertension is defined by a systolic BP ≥ 140 mmHg and/or diastolic BP ≥ 90 mmHg, measured after at least 5 min of rest in the supine position in patients with known neurogenic OH [62].

A distinction is made between the following severity degrees:

o Mild supine hypertension: systolic BP values of 140–159 mmHg or diastolic of 90–99 mmHg;
o Moderate supine hypertension: systolic BP values of 160–179 mmHg or diastolic of 100–109 mmHg;
o Severe supine hypertension: if systolic BP ≥ 180 mmHg or diastolic BP ≥ 110 mmHg.

In addition, patients with cardiovascular autonomic failure often have nocturnal hypertension, which is associated with a loss of the physiological fall in blood pressure (> 10%) during sleep. A distinction is made between two nocturnal blood pressure profiles:

o reduced dipping, characterized by an average nocturnal BP reduction < 10% with respect to daytime values.
o non-dipping OR nocturnal BP rise: when the average BP does not fall or even increases overnight.

**Table:** 

Recommendation	
In individuals with PD and neurogenic OH, a Schellong test with at least a 5-min in the supine position, keeping home BP diaries and 24-h ambulatory BP monitoring should be sought to diagnose supine or nocturnal hypertension	
Level of consensus: 95%, consensus	

#### How to treat nocturnal (supine) hypertension in PD?

For the treatment of nocturnal (supine) hypertension in PD, experts recommend the use of a stepwise approach [100]. Non-pharmacological preventive and behavioral measures should be sought for, including avoidance of the supine position during daytime, patient education with regard to the timing of anti-hypotensive medication intake in case of OH [e.g., avoidance of any pressor agent intake after 4 p.m.], reducing fluid intake in the evening, eating a small meal before bedtime, and sleeping in a 10°–20° head-up tilted position, or steeper if tolerated. If the concomitant OH requires pharmacological treatment, short-acting pressor agents (e.g. midodrine, droxidopa) should be preferred over long-acting ones (e.g. fludrocortisone).

Small-sized studies reported on the efficacy of bedtime administration of transdermal nitroglycerin (0.1–0.2 mg/h) [242], losartan (25–100 mg) [11], eplerenone (50 mg) [10], sildenafil (25 mg), nebivolol (5 mg) [161] and clonidine (100–150 µg) [208] in improving nocturnal hypertension in individuals with primary autonomic failure, including PD. A bedtime administration of short-acting antihypertensive agents (e.g. losartan 25–100 mg, nitroglycerine 0.1–0.2 mg/h transdermal patch overnight, carefully dosed on the individual hemodynamic profile at 24 h ambulatory BP monitoring) can be, therefore, considered in the opinion of the authors of the current manuscript in case of severe nocturnal hypertension, bearing in mind the risk of worsening OH.

**Table:** 

Recommendation	
For the treatment of (nocturnal) supine hypertension in PD, the following stepwise approach should be followed	
1. Non-pharmacological preventive and behavioral measures (see recommendation below)	
2. Switching from long-acting to short-acting pressor agents	
3. Short-acting anti-hypertensive agents at bedtime (see recommendation below)	
Level of consensus: 95%, consensus	
For the prevention and treatment of (nocturnal) supine hypertension in PD, the following non-pharmacological preventive and behavioral measures should be sought	
Avoidance of the supine position during daytime	
Refraining from taking any pressor agent after 4 p.m.	
Reducing fluid intake in the second half of the day (after 18:00)	
Eating a small meal before bedtime	
Sleep in a 10°–20° head-up tilted position	
Level of consensus: 95%, consensus	
For the pharmacological treatment of nocturnal supine hypertension, bedtime administration of clonidine, eplerenone, losartan, nebivolol, nitroglycerin or sildenafil (alphabetically ordered) can be considered	
Level of consensus: 95%, consensus	

#### How to diagnose constipation in PD?

Lower gastrointestinal dysfunction in PD often manifests with problems with passing stools, hard stools, several days without bowel movements, feeling of fullness, and sometimes abdominal pain. Such symptoms may be already present in the prodromal stage of PD [259]. At the time of first diagnosis, constipation is present in 24–63% of PD individuals, depending on the diagnostic criteria used for constipation [219], and its prevalence raises up to 90% with disease progression [251], ultimately impacting on the quality of life of PD individuals, who may notwithstanding tend to underreport it [251].

The guideline on ‘Chronic constipation’ of the German Arbeitsgemeinschaft der Wissenschaftlichen Medizinischen Fachgesellschaften (AWMF) addresses the difficulty of defining constipation in clinical practice, including the setting of PD [3]: already in 1994, Probert et al. pointed out the discrepancy between subjectively reported constipation and consensus definitions, ultimately leading to the wide prevalence ranges found in the literature. One of the main difficulties is that a purely objective definition of constipation, e.g. solely based on stool frequency, does not capture the complexity of symptoms experienced by a large proportion of individuals with chronic constipation. Many patients are, for example, only able to pass stools if pushing hard and applying a major strain, but manage in this way to have daily bowel movements. Likewise, gastrointestinal transit time measurements correlate with stool consistency rather than stool frequency. For this reason, the most recent and internationally acknowledged definition of constipation, i.e. the Rome IV criteria, combines subjective (e.g. heavy straining or incomplete evacuation) and objective parameters (e.g. stool frequency, stool consistency) [146] and was taken over in the German guideline for the diagnosis and treatment of constipation [3].

The diagnostic work-up of individuals with chronic constipation should include a detailed medical history concerning the stool behavior, medication intake including those that may worsen constipation (i.e. opioids, tricyclic antidepressants, calcium-containing antiacids, antihypertensives, antispasmodics, sympathomimetics, and diuretics), concomitant symptoms and diseases that may contribute to constipation. The physical examination should encompass an inspection of the perineal region, a digital rectal examination with evaluation of the sphincter tone at rest and upon contraction, as well as a defecation attempt [3].

Further examinations are not usually recommended unless there are severe symptoms, warning signs and/or pain warranting immediate additional examinations. These are also to be considered in case constipation does not respond to probatory laxative therapy. Additional examinations aim firstly at excluding an obstructive cause and, secondly, at clarifying the pathophysiological substrate of constipation by means of functional examinations. A stool culture is generally not recommended [3].

**Table:** 

Recommendation	
The AWMF criteria should be used to diagnose constipation in individuals with PD	
Chronic constipation is present if the following three criteria have been met for at least 3 months	
(1) ≥ 2 of the following symptoms should be present	
Lumpy or hard stools (Bristol Stool Form Scale 1–2) in > 25% of defecations	
Strong straining in > 25% of defecations	
Subjective incomplete evacuation in > 25% of defecations	
Subjective obstruction in > 25% of defecations	
Manual manoeuvres to facilitate stool expulsion in > 25% of defecations (digital manipulation, pelvic floor support)	
< 3 spontaneous bowel movements per week	
(2) Soft stools rarely occur without the use of laxatives	
(3) The criteria for irritable bowel syndrome are not met	
Level of consensus: 100%, strong consensus	

#### How to treat constipation in PD?

These recommendations largely reflect the German AWMF guideline for treating chronic constipation in the general population [3]. Non-pharmacological measures include a diet rich in fibers and a fluid intake of 1.5–2 L per day. Any additional fluid intake has no further effect on constipation [3]. Regular exercise should be also aimed for, while physical activity beyond the appropriate level for the respective age does not result in an increased therapeutic effect [3]. On the other hand, the relationship between constipation and physical inactivity is well acknowledged. A diet rich in fibers can result in abdominal disturbances and even pain at the beginning, but these usually diminish with prolonged administration. Especially in elderly individuals, the individual tolerance of natural dietary fibers should be taken into account and attention should be paid to sufficient fluid intake. Soluble fibers can be tried as an alternative, but it should be born in mind that the prolonged transit time typically observed in PD individuals with constipation may lower the overall tolerance to an increased dietary fibers supply [3].

Small studies showed that abdominal massages may have positive effects on constipation on PD individuals [145], especially if following a tensegrity technique, which is based on balancing the tension of muscles, fascia and ligaments [108].

Macrogol (i.e., polyethylene glycol 3350 or 4000, PEG, 13–26 g o.i.d.) is an osmotic laxative and the pharmacological option of first choice against constipation in PD. A meta-analysis [119] also showed that PEG is superior to lactulose in terms of efficacy (i.e., stool frequency, stool form, relief of abdominal pain) and safety profile. For an improved effect, macrogol should be administered regularly, not on demand, with sufficient fluids and the dosage should be adjusted individually, informing patients that even at increased dosages, macrogol remains barely absorbable [3]. Other pharmacological options include prucalopride (1–2 mg o.i.d.) or a combination of bisacodyl (5–10 mg o.i.d.) and sodium picosulphate (5–10 mg o.i.d.), administered in the evening, these latter also barely absorbed from the gastrointestinal tract. Comparative studies between macrogol and bisacodyl/sodium picosulphate are not available, but, according to a network meta-analysis, bisacodyl/sodium picosulphate appeared to be more effective than PEG [131]. Such substances have been indeed often used as rescue medications in interventional trials for constipation [3].

Fermented milk containing probiotic bacterial strains proved efficacious in increasing the frequency of defecations, improving the stool consistency and reducing the necessity of laxatives intake in PD individuals [14]. Bacterial strains contained in probiotic capsules also showed a significant increase in the number of weekly defecations and an improved quality of life in PD individuals in past interventional studies [224]. It has been postulated that probiotics may also influence PD motor and non-motor symptoms such as cognition, depression and psychosis, but long-term studies are missing at present [225].

Rectal evacuation aids are available for severe constipation, including bisacodyl-cellulose or CO_2_-releasing suppositories.

**Table:** 

Recommendation	
For the treatment of constipation in PD individuals, including geriatric ones, the AWMF guidelines for "Chronic constipation" should be followed. The most important general recommendations are (see the original guideline for detailed recommendations [3])	
Secure a fluid intake of 1.5–2 L per day	
Avoid physical inactivity	
Avoid suppressing the urge to defecate	
Increase the amount of dietary fibers. In geriatric patients, the individual tolerance of natural dietary fibers should be taken into account and attention should be paid to sufficient fluid intake. Soluble dietary fibers can be tried as an alternative. If gastrointestinal side effects occur, the intake of dietary fibre should be reduced and other measures to treat constipation prioritized	
Macrogol should be used as the drug of first choice to treat constipation in PD and there is no reason to limit the period of use	
Sodium picosulphate, bisacodyl, and prucalopride represent alternative pharmacological options in individuals for whom general measures and macrogol were not sufficiently effective or poorly tolerated	
Further pharmacological options include antraquinones, linaclotide, plecanatide, sugar and sugar alcohols (lactulose, lactitol, sorbitol and lactose)	
Non-pharmacological measures for treating chronic constipation in the general population include biofeedback, pelvic floor training and acupuncture procedures (acupuncture, acupressure, moxibustion, electro-acupuncture), with limited evidence in the specific setting of PD	
In PD, probiotics, prebiotics and synbiotics (without strain-specific recommendations), as well as abdominal massage (especially if following the tensegrity technique) were shown to improve constipation and can be recommended as additional measures	
Level of consensus: 100%, strong consensus	

### Pain

#### How does pain in individuals with PD differ from pain in individuals without PD in terms of frequency, impairment of quality of life and risk factors?

Pain in PD is common [197], predominantly chronic [30] and has a negative impact on quality of life [130, 137, 210]. The prevalence of pain in PD individuals ranges from 68 to 95%, depending on the characteristics of the examined population, applied study design and assessed pain types [29]. Pain can occur early in disease course or even precede a PD diagnosis [124], but with disease progression, the majority of PD individuals will ultimately experience painful sensations [30, 137, 154, 237]. Female gender, dyskinesias, postural instability, motor complications and depression have been found to predict painful sensations in PD individuals [30]. The prevalence of pain in PD is likely higher than in the general population, in which 50–80% of individuals over 65 years of age reported occasional or regular pain.

**Table:** 

Recommendation	
Patients with PD should be asked about pain, because pain is very common in PD and has a negative impact on quality of life	
Level of consensus: 100%, strong consensus	

#### Which subtypes of pain can occur in PD?

There is no general and unambiguous pain classification system applicable to PD. For a long time, the Ford pain classification was most commonly used in PD settings. This distinguishes between musculoskeletal, radicular/neuropathic, dystonia-related and akathisia-related, as well as central pain [73, 74]. Wasner and Deuschl subsequently proposed an improved four-tier taxonomy for classifying pain in PD. This taxonomy firstly distinguishes between nociceptive, neuropathic and other pain types into different categories, which are further divided into subcategories [252]. A recently proposed pain classification system for PD (PD-PCS) aims at differentiating PD-related from PD-unrelated pain using four questions, followed by additional distinctions based on the International Association for the Study of Pain (IASP) criteria. In addition to the well-known categories of neuropathic and nociceptive pain, the IASP criteria introduced the concept of nociplastic pain to describe persistent pain that arises from altered nociception, despite no clear evidence of actual or threatened tissue damage causing the activation of peripheral nociceptors, or evidence for disease or lesion of the somatosensory system causing the pain [165]. The term nociplastic pain was first used in 2019 to describe central pain and restless legs syndrome in PD [135], but has not been used in studies on pain in PD yet. The PD-PCS system has been however validated considering the presence of nociplastic pain in PD [154]. According to the PD-PCS classification, PD-related pain is defined as a pain that occurs in association with motor symptoms, responds to dopaminergic medication, or is exacerbated by PD motor fluctuations, including OFF-phenomena. Such pain can be nociceptive, nociplastic or neuropathic in nature [153, 154] and is to be distinguished from pain that occurs independently from PD. To this end, pain that increases with increased rigidity and improves with dopaminergic medication intake is to be considered as PD-related or PD-dependent. Pain that develops with motor fluctuations and is often associated with dystonia, as well as visceral pain that PD individuals may describe as internal or localized in the belly, should be likewise considered as PD-related. Unpleasant and sometimes painful sensations in the extremities such as edema or in connection with restlessness, especially overnight, such as in the case of restless legs syndrome, should also be inquired about. Examples of frequent pain causes that are less related to PD include pain due to articular or vertebral osteodegenerative changes, even though these may also occasionally improve with dopaminergic medications.

There is consensus that musculoskeletal pain is the most common type of pain in PD. Several epidemiological studies reported that this nociceptive form of pain occurs in 40–80% of PD individuals [41, 137, 189, 237]. Neuropathic pain has been reported in 10–30% of PD individuals [137, 152], while Mylius et al. found a 22% frequency of PD-related nociplastic pain, laying between nociceptive (55%) and neuropathic (16%) pain [153, 154].

**Table:** 

Recommendation	
In PD individuals, a distinction should be made between PD-related and PD-unrelated pain	
When it comes to PD-associated pain, a distinction should be applied between nociceptive, nociplastic and neuropathic pain	
Level of consensus: 100%, strong consensus	

#### Which scales and questionnaires are suitable for assessing pain in PD?

Identifying pain in PD is important, because it is frequent and has a major impact on the quality of life of affected individuals. Individuals with PD may not see a connection between painful sensations and their PD diagnosis and therefore often do not report it to their treating neurologist. This could be one of the reasons why neurologists often play a subordinate role in the treatment of pain compared to orthopedists and general practitioners [30]. PD individuals should be therefore specifically asked about painful sensations during neurological consultations, taking into account the following pain subtypes: musculoskeletal, neuropathic, fluctuation-dependent, dystonic, visceral (often referred to as deep internal) and pain in the extremities accompanied by edema or restless legs syndrome. Pain documentation should be ideally carried out in a structured way using scales or questionnaires.

Most PD scales unfortunately only have a marginal focus on pain. In the Unified Parkinson Disease Rating Scale (UPDRS), item 17 asks about “sensory complaints as a result of parkinsonism” and item 34 asks to what extent dyskinesias are painful. The newer Movement Disorder Society UPDRS (MDS-UPRDS) also asks little about pain (Item 1.9: Pain and other sensations; Item 4.6: Painful dystonia in the off state).

In the Non-Motor Symptom Scale (NMSS), item 27 asks about pain that cannot be explained by other illnesses. In the newer MDS Non-Motor Rating Scale (MDS-NMS), pain is assessed more in detail with a division into muscle/joint/back pain versus deep or dull body pain versus dystonia-related pain or pain from other causes such as nocturnal or facial pain. The MDS-NMS also documents pain severity and to what extent pain severity changes in response to motor fluctuations.

At present, two pain assessment tools have been specifically developed and validated in PD settings: the King’s Parkinson’s disease Pain Questionnaire and related Scale (KPPQ and KPPS) [41, 103], and the Parkinson’s disease pain classification system (PD-PCS) [137, 153]. In the KPPS, pain is initially divided into 7 categories (musculoskeletal, pain deep in the body, fluctuation-dependent pain, nocturnal pain, facial pain, pain caused by edema, radicular pain), and subsequently further characterized based on 14 subcategories. In the PD-PCS, an association between pain and PD is inquired before further categorizations are operated. An association between pain and PD is assumed, if at least one of the following criteria applies: pain has a temporal association with the development of parkinsonism, increases if rigidity, bradykinesia or tremor worsen, is associated with motor fluctuations and/or decreases under dopaminergic medication intake.

**Table:** 

Recommendation	
The following scales/questionnaires should be used to assess pain in Parkinson's patients	
1. Pain assessment and pain categorization: Parkison’s disease pain classification system (PD-PCS)	
2. Pain assessment and quantification: King’s Parkinson’s disease Pain Scale (KPPS)	
Level of consensus: 100%, strong consensus	

#### How to treat pain in PD?

In clinical practice, an attempt should be made to classify pain according to its etiology (mechanism-based classification approach) and this should in turn guide the therapeutic strategy.

**Fig. 1:**
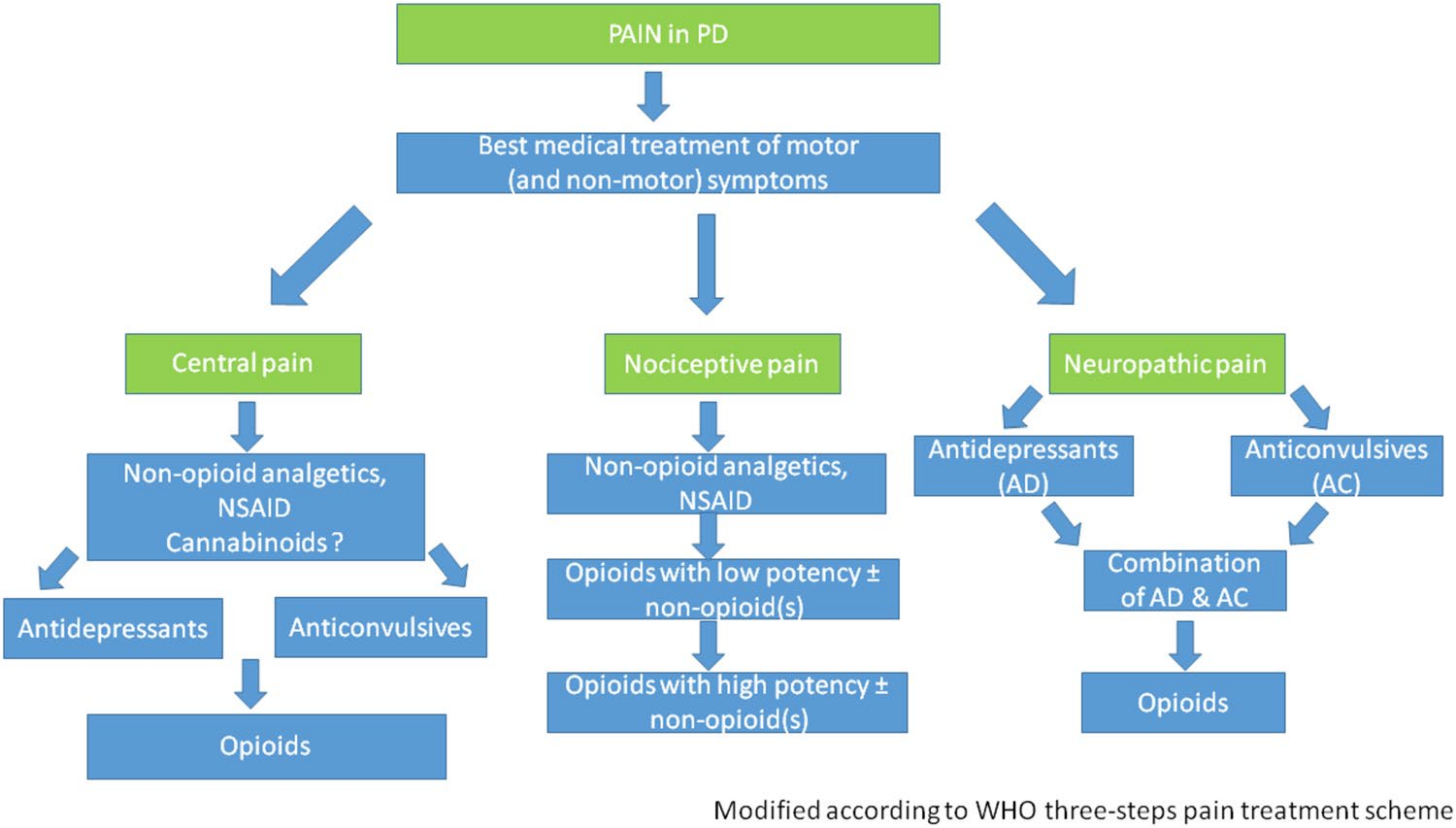
Algorithm for treating pain in PD. Reproduced with permission from Journal of Parkinson’s disease, vol. 10, no. s1, pp. S37–S48, 2020

Optimizing dopaminergic and other anti-parkinsonian medications is essential to improve PD-related pain. This also includes the treatment of painful dystonia and restless legs syndrome that fall under the category of nociplastic PD-related pain. Regardless of whether PD-related or not, nociceptive pain should be treated according to the WHO 3-step pain treatment ladder, while the treatment of neuropathic pain should follow the guidelines for neuropathic pain in other diseases. An algorithm for treating pain in PD individuals is proposed in Fig. 1 [29].

There is no uniform approach to the treatment of pain in PD, because the painful sensation experienced by single individuals can vary substantially in terms of quality and quantity, and because there are no comparative trials between different approaches. In available controlled and uncontrolled trials with different compounds, pain was usually not the primary endpoint, no analysis was performed according to different pain subtypes, and a placebo or a standard of care was usually used as a control. Pain-relieving effects have been demonstrated for some antiparkinsonian medications, including pramipexole, rotigotine and safinamide (summarized in [29, 106]). Advanced PD therapeutic options such as apomorphine or levodopa pump-therapies or deep brain stimulation of the subthalamic nucleus could also improve specific pain subtypes [29]. It is conceivable that part of the pain reduction achieved with classic antiparkinsonian drugs is due to an improved dopaminergic stimulation or a reduction in motor fluctuations, so that pain treatment strategies may entail an adjustment of levodopa dosages or add-on of COMT inhibitors.

Table 1 provides an overview of the randomized control trials for the treatment of pain in PD with antiparkinsonian medications and opiates [29], as well of the studies with other compounds providing lower levels of evidence for pharmacological treatment of pain in PD individuals. Additionally to the consensus guidelines, local botulinum toxin application might be considered in special cases of pain refractory to maximized oral or transdermal drug therapy [29].

**Table 1:** Studies investigating the effects of antiparkinsonian medications, opioids and other drugs on pain in individuals with PD

Treatment	Study design	Applied pain scales	No. of patients versus controls	Results Means with (SD), (range) or (CI)
*Randomized controlled trials*				
Pramipexole [16]	RCT	VAS-P	*N* = 138 versus 148	− 3,3 (− 8.9 to 0.3) versus − 2.4 (− 8.9 to 2.6) Group comparison − 1.3 (− 3.3 to 0.8) *p* = 0.19
Safinamide [36]	RCT (post hoc)	PDQ-39	*N* = 440 versus 438	100 mg daily dosage: 23.6% reduction of the number of concomitant pain treatments [95% confidence interval (CI): 41.1%, 1.0%; *p* = 0.0421] PDQ-39: item 37 (− 0.26 vs. − 0.07; *p* = 0.0009), item 38 (0,19 vs. 0.1; *p* = 0.1585), item 39 (− 0.18 vs. − 0.03; *p* = 0.0060)
Entacapone [162]	RCT	PDQ-39	*N* = 281 vs. 274	(0.04) versus 0.1 (0.04); *p* = 0.9. SMD = 1.81, *p* < 0.0001
Oxycodone [233]	RCT	KPPS	*N* = 88 versus 106	5.0 (95% CI 4.5 to 5.5) versus 5.6 (5.1 to 6.0) Difference − 0.6, 95% CI − 1.3–0.0; *p* = 0.058)
Pardoprunox [185]	RCT (post-hoc)	VAS-P	*N* = 140 versus 133	During OFF time − 2.2 (2.7) versus − 1.0 (2.7), no p-values given During ON time − 2.3 (2.8) versus − 0.5 (3.0), no p-values given
Rotigotine [109, 234]	RCT RCT (post-hoc)	Likert pain scale Likert pain scale	*N* = 178 versus 89 *N* = 178 versus 89	− 0.9 (SD 2.2) versus − 0.1 (SD 2.3), *p* = 0.004 Difference − 0.77 [− 1.28 to − 0.25] 'Any pain': − 0.88 [95% CI − 1.56, − 0.19], p = 0.013) 'Moderate-to-severe' pain: (− 1.38 [− 2.44, − 0.31], p = 0.012 UPDRS III or PDSS-2 responders showed greater improvement in pain than non-responders
*Studies with anti-Parkinsonian medications with lower level of evidence (III or IV)*				
Rotigotine [109, 231]	RECOVER—Post-hoc analysis NEUPAD—Non-interventional multicenter observational study over 4 weeks	Likert pain scaleerman Pain Questionnaire	*N* = 178 versus 89 *N* = 70	Predictors for treatment response: higher pain level, improvement in UPDRS III and PDSS-2 with rotigotine treatment Non-significant reduction in the affective dimension of pain (probably dependent on motor improvement)
Safinamide [36, 37, 82, 195]	Post-hoc analysis of studies 016 and SETTLE (placebo-controlled) over 24 weeks Post-hoc analysis of study 018 (placebo-controlled) over 2 years Open-label prospective single center study over 12 weeks Open-label prospective multicenter study over 6 months (SAFINOMOTOR; Phase IV)	PDQ-39 PDQ-39 KPPS, BPI KPPS, PDQ-39	Study 016: *N* = 224 versus 222 Study SETTLE: *N* = 274 versus 275 N = 180 versus 175 *N* = 13 *N* = 50	Improvement in 2 out of 3 domains of physical limitations (musculoskeletal and neuropathic pain) in the PDQ-39 Reduced frequency of concomitant pain therapies [100mg/d] [36, 37] Significant improvement in PDQ-39 items 37 (painful cramps or spasm) and 39 (unpleasantly hot or cold) Significantly reduced number of concomitant pain medication use versus baseline and versus placebo Significant improvement in KPPS and BPI Intensity and Interference Significant improvement of PDQ-39, Item 8 (pain and discomfort) -Significant improvement on the KPPS, − 43.6% (p < 0.0001)
Levodopa-Carbidopa intestinal gel [8]	GLORIA; international postmarketing study	UPDRS, NMSS, PDQ-8, EQ-5D	*N* = 375	-Less painful dyskinesia (after 12 months of treatment) and muscle cramps (after 6 months)
*Studies with other pain medications*				
Non-opioid analgesics	No studies available			
Antidepressants				
Duloxetine [53]	Open-label, 6 week	VAS, BPI, Mc Gill short form, PDQ-39, UPDRS-III	*N* = 20	Reduction of central and peripheral neuropathic pain
Antiepileptics	With the exception of gabapentin, no studies addressing pain in PD, data for efficacy on neuropathic pain driven from other populations			
Gabapentine [163, 238]	Double-blind, placebo-controlled, crossover trial Double-blind, placebo-controlled, crossover trial, 4 weeks of treatment	UPDRS UPDRS	*N* = 19 *N* = 20	Total UPDRS improvement with gabapentine compared with placebo (*p* = 0.0005) UPDRS III improvement with gabapentine compared with placebo (*p* < 0.001)
Botulinum toxin				
Incobotulinum toxin A [186]	Double-blind randomized trial 6 and 18 weeks post-injection	CGI, Dystonia severity and associated pain	*N* = 45	Reduced dystonic plantar flection and associated pain in BoNT group versus baseline but not versus placebo
Onabotulinumtoxine A [28]	Double-blind, randomized crossover trial 4 and 12 weeks post-injection	NRS	*N* = 12	Reduced global limb pain in BoNT group versus baseline but not versus placebo
Cannabinoids				
Marijuana [128, 209]	Two open-label, uncontrolled observational studies	UPDRS, present pain intensity scale, PRI, VAS, Mc Gill short-form	*N* = 22 and *N* = 20	Pain reduction measured with different scores 30 min after smoking 0.5–1.0 g Marijuana
Nabilone (synthetic THC analogue) [170]	Double-blind, placebo-controlled, randomized 4-weeks trial	KPPS, VAS	*N* = 38 (19 nabilone, 19 placebo)	Pain improvement
Various cannabinoids [68, 260]	Two question-naire-based uncontrolled national studies in the PD community	Ad-hoc questionnaires	*N* = 113 (Germany) *N* = 261 (USA)	Pain reported in 57 PD individuals with improvement in *N* = 25 (44%) Pain reported in 88 patients with considerable or moderate improvement of severity and frequency in *N* = 63 (72%) and *N* = 56 (64%), respectively

*BoNT* Botulinum Neurotoxin, *BPI* Brief Pain Inventory, *CGI* Clinical Global Impression, *CI* confidence interval, *EQ-5D* European Quality of Life-5 Domains, *KPPS* King’s Parkinson’s disease Pain Scale, *NMSS* Non-Motor Symptom Scale, *NRS* Numeric Rating Scale, *PDQ-39/8* Parkinson’s disease questionnaire-39/8 items, *PDSS* Parkinson’s disease sleep scale, *PRI* Pain rating Index, *RCT* randomized-controlled trial, *SD* standard deviation, *VAS-P* visual analogic scale-pain, *UPDRS* Unified Parkinson’s disease rating scale

**Table:** 

Recommendation	
Severe chronic pain in PD should be treated in a multimodal, interdisciplinary manner, like other pain disorders Pain treatment should be guided by the etiology of pain The basis of treatment is to optimize the antiparkinsonian medication schedule, whereby levodopa, dopamine agonists, COMT inhibitors or safinamide can be used to treat dopamine-dependent pain Nociceptive pain should be treated according to the 3-step WHO regimen Neuropathic pain should be treated with anticonvulsants and/or antidepressants according to the guidelines for the treatment of neuropathic pain, with preference given to gabapentin and/or duloxetine (particularly in case of concurrent depression) If pain is severe, treatment with oxycodone/naloxone may be considered	
Level of consensus: 100%, strong consensus	

### Sleep

#### Which sleep disturbances may occur in PD?

Individuals with PD report insomnia with difficulties falling asleep and maintaining sleep, pain, involuntary movements and restlessness of limbs, worsening of motor symptoms such as nocturnal akinesia, rigidity and/or resting tremor, nocturia, nocturnal behavioral disorders, including REM-sleep behavior disorders (RBD) and non-REM parasomnias, anxiety, non-restorative sleep with fatigue and/or daytime sleepiness, and breathing disorders [38, 207, 218]. The etiology of these symptoms may be classified into comorbid primary sleep disorders, motor and non-motor symptoms of PD, coexistence and influence of neuropsychiatric disturbances, autonomic dysfunction, side-effects of PD medications and the impact of neurodegeneration itself on cerebral sleep regulating centers [38, 134, 218, 244, 266, 268]. A bidirectional relationship between sleep disorders and disease progression has been postulated [266].

##### Comorbid primary sleep disorders

Sleep disorders listed as an independent diagnosis in the ICSD-3 (International Classification of Sleep Disorders) are considered primary sleep disorders. These entail Restless Legs Syndrom (RLS), sleep disordered breathing (SDB), and parasomnias, including RBD, which occurs in PD as well as in other α-synucleinopathies:

- RLS: The overlap between nocturnal restlessness in PD, for instance in the context of wearing-off phenomena, and RLS may complicate the diagnostic differentiation [38]. Several studies investigating the prevalence of RLS in PD came to contradictory results [21, 85], whereas a meta-analysis concluded on an overall increase in RLS prevalence in PD [5]. Due to the distinctly different pathophysiology, RLS is interpreted as a comorbidity in PD, with newly diagnosed PD individuals initially showing the same prevalence of RLS as non-PD subjects. The prevalence of RLS increases with PD progression and dopaminergic daily dosage. Whether individuals with RLS have a higher risk of developing PD is a matter of debate [201]. A prospective longitudinal questionnaire study showed an elevated incidence of PD in individuals with severe RLS [222].
- SDB: Obstructive and central sleep apneas with recurring oxygen desaturation lead to sleep fragmentation, nocturia, daytime sleepiness and increased cardiovascular risk. Data on the prevalence and severity of SDB in PD patients are controversial [218]. However, a recent meta-analysis showed that PD patients with obstructive sleep apnea (OSA) suffer more severe motor and cognitive symptoms [56]. Although the occurrence of excessive daytime sleepiness in PD does not solely depend on the severity of SDB and the risk for a severe form of SDB does not seem to be elevated in PD patients, an individualized diagnostic work-up and therapeutic approach is recommended to minimize the risk for concomitant cardiovascular disease [218].

##### PD-related symptoms

- Nocturnal „wearing-off“: Actigraphy studies show a decrease in spontaneous movements towards the morning in PD patients, suggesting a dopaminergic deficit [121, 215] with recurrence of motor symptoms such as tremor, rigidity and akinesia with patients‘ inability to turn in or get out of bed, increased nocturia, as well as painful dystonic cramps in early-morning OFFs.
- Wearing-off phenomena may include neuropsychiatric symptoms, such as anxiety and depression.
- Autonomic failure: especially at advanced PD stages, autonomic features such as nocturia, OH, constipation, reflux, pain and sweating may cause or worsen sleep fragmentation [24].

##### Adverse effects of pharmacotherapy

Dopaminergic medication, especially dopamine agonists are known to induce daytime sleepiness and sleep attacks [79]. This adverse effect appears dose-dependent: high dosages increase sleep fragmentation and nocturnal wakefulness and reduce the amount of slow-wave sleep, whereas low dosages improve sleep quality and reduce awakenings after sleep onset [226]. Amantadine is also known to increase nocturnal wakefulness and reduce slow-wave sleep [230]. In case of sleep disturbances, the beneficial and adverse effect of these medications needs to be balanced. Hallucinations, psychosis and impulse control disorders likewise have a negative impact on objective and subjective sleep quality [120, 190, 218, 269].

##### Neurodegenerative alterations in sleep-regulating networks

- RBD: RBD is a recognized possible prodromal feature of PD and other α-synucleinopathies, as well as a progression marker in clinically manifest PD [19, 157, 164] that may lead to nocturnal disruptive behaviors, stressing both PD individuals and their bed partners. A careful differentiation of other abnormal nighttime behaviors by means of video-polysomnography (VPSG) is essential.
- Changes in sleep micro-and macrostructure: with disease progression, a progressive deconstruction of normal sleep macrostructure with a loss of slow-wave sleep, as well as changes in the sleep microstructure are observed [134, 177, 266, 268]. The loss of delta waves and sleep spindle reduction has been associated clinically with faster cognitive decline [52], likely due to a disturbed sleep-dependent restorative function for metabolic homeostasis. The destruction of physiologic sleep architecture and loss of slow-wave sleep also impairs the glymphatic system—a glial-dependent perivascular network with an important role in the clearance of neurotoxic protein waste, such as beta-amyloid and aggregated α-synuclein. These factors may contribute to the malfunction of cerebral sleep regulating centers and possibly enhance the neurodegeneration cascade [25, 202, 266]. Clinically, patients complain about difficulties with sleep maintenance, early morning awakenings, non-restorative sleep, and in some cases problems falling asleep.
- Impairment of circadian rhythm: changes in the expression of clock genes have been found in PD individuals. These in turns impair the circadian rhythm of kidney function, blood pressure regulation, as well as of ADH, cortisol and melatonin secretion. A progressive loss of orexinergic neurons in the prefrontal cortex and hypothalamus has been found to result in a significant fall of orexin and melatonin levels [94, 134, 247, 261]. Again, a bidirectional relationship between impaired circadian rhythm and neurodegeneration has been hypothesized [94].

**Table:** 

Recommendation	
Sleep disturbances in PD are frequent, manifest with multiple symptoms and should be considered in all diagnostic and therapeutic decisions. Frequent sleep disturbances in PD include insomnia, pain, RLS, nocturnal akinesia, tremor, rigidity, nocturia, parasomnias, nightmares, SDB and RBD	
Level of consensus: 100%, strong consensus	

#### How to screen for sleep disturbances in PD?

PD individuals seldom report sleep problems spontaneously. Besides specifically addressing individual sleep history and questioning bed partners and caregivers, validated questionnaires help to screen for sleep disturbances and estimate the overall sleep satisfaction. Sleep diaries and morning/evening protocols may also be useful.

Regarding screening questionnaires, an expert consensus conference endorsed by the Movement Disorder Society [92] recommended to use the Parkinson’s Disease Sleep Scale (PDSS-2), the Pittsburgh Sleep Quality Index (PSQI) and the Scale for Outcomes in Parkinson’s Disease-Sleep (SCOPA-S). The PSQI was not validated in PD [31]. The SCOPA-S was validated for PD and captures PD-specific nocturnal disturbances as well as daytime sleepiness during the last 4 weeks, but does not ask for potential causes [136, 138]. The PDSS-2 was validated as a numeric scale [235] based on the original PDSS as a visual analogous scale [40]: 15 questions cover overall sleep quality and its restorative effect, as well as PD specific nocturnal problems like nocturnal akinesia, nocturia, nightmares, restlessness, nocturnal pain, and nocturnal breathing problems during the last week, thus creating a profile of specific complaints and potential causes. A cut-off of > 18 points has been validated as indicative of a sleep disturbance and warranting an additional diagnostic work-up with vPSG [151]. Sleep therapeutic studies usually utilize the PDSS-2, as it enables simplified comparisons and follow-up [138]. There are no comparative studies between the three scales.

**Table:** 

Recommendation	
The PDSS-2 should be used as a screening instrument for sleep disturbances in PD: the numeric scale simplifies comparisons. The evaluation of reported complaints and symptoms may point towards potential causes of the sleep disturbance	
Level of consensus: 100%, strong consensus	

#### How to diagnose sleep disturbances in PD?

If patient and/or caregiver reports, sleep diaries and questionnaires do not clarify the nature of the sleep disturbance, objective measurements are needed to document the course of nocturnal sleep including motor phenomena and behaviors.

- Actigraphy: Around-the-clock activity and resting states are registered in the home environment for a longer period of time, thus enabling an estimation of total sleep time, sleep efficiency, sleep latency and sleep fragmentation, as well as screening for circadian rhythm disorders. The accuracy of actigraphy deteriorates with disease progression [132, 143].
- Cardiorespiratory polygraphy additionally collects data on breathing, cardiac activity and oxygen levels. It is mainly used to screen for SDB in an outpatient setting.
- vPSG: a laboratory-based vPSG currently represents the diagnostic gold standard. Additional EEG and EMG registrations enable sleep staging and a more in-depth analysis of sleep structure, sleep fragmentation and sleep efficiency. Synchronized video-registration of nocturnal behaviors and motor phenomena helps to clarify nocturnal disturbances that are not satisfactorily explained by patient history, bed partner/caregiver report or the above mentioned screening instruments. According to the International Classification of Sleep Disorders (ICSD-3), vPSG is currently mandatory for a diagnosis of RBD. Future technologies may close the gap between outpatient screening and sleep laboratory settings, as pointed out by recent data on the actigraphy-based detection of RBD [184].

**Table:** 

Recommendation	
In case of sleep disorders that require treatment, objective assessments such as actigraphy, cardiorespiratory polygraphy and vPSG can be recommended, especially if first therapeutic measures, such as optimizing medication, did not prove beneficial Actigraphy may be considered to estimate total sleep time, sleep efficiency and wakefulness in PD individuals, facilitating the sleep rhythm evaluation over a longer period of time. The accuracy of actigraphy however deteriorates with disease progression vPSG is the gold standard for identifying sleep disturbances and a prerequisite for diagnosing RBD. If severe SDB is suspected, polygraphy or vPSG should be recommended	
Level of consensus: 100%, strong consensus	

#### How to treat sleep disturbances in PD?

The diverse etiology and clinical manifestations of sleep disorders in PD imply an individualized therapeutic approach addressing symptoms based on their sleep-related diagnosis.

##### Comorbid primary sleep disorders

- RLS: there are no randomized controlled trials for the treatment of RLS in the specific setting of PD. Currently valid German guidelines for RLS (without comorbid PD) treatment [91] recommend a work-up of iron metabolism, aiming at high normal serum levels of ferritine and transferrin saturation. RLS-aggravating medications, such as some antidepressants should be tapered and discontinued if possible. If these measures are insufficient, optimizing dopaminergic medication according to the individual risk profile, as well as alpha-2-delta ligands or opioids may be considered [38, 201].
- SDB: Treatment for SDB in PD individuals should follow the current guidelines for treating sleep apnea in the general population [38, 144, 201, 226]: in case of obesity, weight reduction and nocturnal positive pressure ventilation—either as CPAP or BiPAP—are recommended. A randomized controlled study showed that CPAP therapy for PD-associated SDB reduces the apnea–hypopnea index and shortens sleep latency [155]. However, therapy adherence in PD individuals is often quite low. Both PD individuals and their caregivers may feel encumbered by the ventilation device, so that an individual benefit assessment seems necessary.

##### PD-related symptoms

- Nocturnal wearing off: treatment should focus on adapting dopaminergic therapy [201, 226]. Long-acting dopamine agonists or slow-release levodopa or pump therapy may be considered, with re-evaluation of sleep quality. In case of increased daytime sleepiness, dopaminergic therapy might have to be reduced, for example with switch between different dopamine agonists or a change to levodopa. In case of persisting sleep disturbances, further diagnostic work-up by vPSG may be considered to exclude SBD, among others.

##### Neurodegenerative alterations in sleep-regulating networks

- RBD: therapeutic guidelines based on high evidence levels do not exist due to difficulties in defining adequate assessment methods. Expert consensus calls for a safe sleep environment and recommends the use of clonazepam, melatonin or rivastigmine [245]. Adverse effects of clonazepam on balance and alertness must be considered, especially in elderly patients. An analysis of all clinical and scientific evidence on the treatment of RBD published in 2022 shows the most positive results for clonazepam (dosages used in comparative studies: 0,125–3 mg) and melatonin (dosages used in comparative studies: 2–9 mg) [84]; therefore, these substances are regarded as first choice medication.
- Impairment of circadian rhythm: the pathophysiological substrate of insomnia associated with alterations of circadian rhythms provides the rationale for melatonin administration in PD. A multicentre, randomized double-blind, placebo-controlled study indeed demonstrated the safety and efficacy of 2 mg slow-release melatonin [2], if assessed by means of the PSQI, as a questionnaire non-specific for PD, the Non-Motor-Symptom Scale (NMSS) and PDQ39, while another study had negative results [50]. Due to the favorable safety profile, the updated MDS evidence-based recommendations [204] regards the use of melatonin as potentially useful and therefore justified for treating circadian rhythm disorders in PD. Non-pharmaceutical measures include bright light application, which showed positive effects on sleep quality and diurnal physical activity [139, 246], general advice on sleep hygiene [201], as well as enforced physical training [46]. A randomized controlled study showed a significant improvement of sleep quality and efficiency under intensive physical training compared to sleep hygiene measures alone [46].
- Changes in micro- and macrostructure of sleep: after excluding other sleep disorders like RLS, SDB, nocturnal wearing-off or depression, a pharmacological treatment may be considered following current German guidelines for treating insomnia in neurological disorders [143]. Eszopiclon (1 mg), doxepin (25 mg), zolpidem (5 mg), trazodone (50 mg), melatonin (2 mg) as well as the antipsychotic drug pimavanserin and the antidepressants venlafaxin (37,5 mg) and nortryptilin (25 mg, both to be considered in case of co-existing depression) may be tried, despite insufficient evidence. Based on a randomized controlled study in non-PD insomniacs [107], the antidepressant mirtazapin may be also recommended in doses of 7.5–15 mg. In any case, pharmacological treatment should be started with the lowest possible dosage. Further adjustments may be required on an individual basis and side effects should be carefully monitored at follow-up. To date, no sleep-restorative therapies are available.

**Table:** 

Recommendation	
Comorbid sleep disorders such as RLS or SDB should be treated according to their specific guidelines If motor or non-motor dopaminergic fluctuations are responsible for the sleep disturbance, dopaminergic therapy should be adjusted accordingly RBD should be treated by creating a safe sleep environment. Clonazepam and/or melatonin may be considered, but potential side effects need to be taken into account After exclusion of medication side effects and/or a primary sleep disorder such as SDB or otherwise treatable causes, insomnia and circadian rhythm disorders can be treated by sleep hygiene, intensive physical training and bright light therapy. Eszopiclon, doxepin, zolpidem, trazodone, melatonin, venlafaxin (in case of comorbid depression), nortryptilin or mirtazapin may be considered for insomnia, despite insufficient evidence (95% consensus)	
Level of consensus: 95%, consensus	

#### How effective are slow-release levodopa preparations or dopamine agonists for treating nocturnal PD motor symptoms?

Whenever PD individuals complain of the nocturnal recurrence of PD symptoms like the inability to turn in or get out of bed, off-dystonic cramps, or the inability to fall asleep again because of tremor worsening, the application of long-acting dopaminergic agents overnight should be considered. To date, the transdermal application of rotigotine is the best investigated dopaminergic treatment option: randomized, controlled trials showed a significant improvement of sleep disturbances [234], with increased sleep efficiency, shortened sleep latency and reduced nocturnal awakenings under rotigotine treatment [172]. Pramipexole was also effective, and ropinirole proved non-inferior [257]. An open-label study showed improvements on PDSS-2 scorings by combining levodopa/carbidopa with entacapon [168]. PD patients on pump-based continuous delivery of dopaminergic medication will benefit from extending the pump running time through the night. Both an extension of daytime subcutaneous apomorphine infusion [69] and isolated nocturnal applications [48] led to a significant improvement of sleep quality. Levodopa/carbidopa intestinal gel instillation via PEG/PEJ through the night also proved beneficial on subjective sleep quality and daytime sleepiness [39, 49, 216]. Of note, dopamine agonists may cause daytime sleepiness and sleep attacks in a dose-dependent manner. Overnight, dopamine-agonists at high dosages were shown to induce nocturnal wakefulness, increase sleep fragmentation and reduce slow-wave sleep, whereas lower dosages reduced nocturnal wakefulness and improved sleep quality [226].

A recent overview [270] described a positive effect of deep brain stimulation on sleep latency and fragmentation. Whether this is due to a direct effect of the stimulation or rather to the postoperatively reduction of dopaminergic medications remains unclear.

It should be kept in mind that many substances successfully used in PD pharmacotherapy have never been tested in trials with sleep disorders as primary endpoint. This is for example the case of slow-release levodopa formulations. The clinical experience, however, underlines its therapeutic benefit, especially when dopamine agonists are contraindicated.

**Table:** 

Recommendation	
For sleep disturbances due to a nocturnal dopaminergic deficit, long-acting dopamine agonists such as rotigotine can be recommended Slow-release levodopa may be offered as an alternative Patients on pump therapy due to PD fluctuations may be offered a nocturnal extension of the pump running time	
Level of consensus: 100%, strong consensus	

#### How to screen for excessive daytime sleepiness in PD?

Daytime sleepiness and sleep attacks show an increased prevalence in PD individuals, representing a major health hazard and impacting on the disease burden, daytime activities, social interactions, ability to drive and, overall, quality of life [6, 214, 262]. Although the propensity to sleep during the day may be an early symptom of PD [80], excessive daytime sleepiness increases with disease progression [6, 211] and may predict mortality [173]. Fatigue needs to be differentiated from daytime sleepiness. A fatigued person may feel tired, prematurely exhausted, but will not unintentionally fall asleep during resting episodes. PD individuals with excessive daytime sleepiness may, by contrast, frequently fall asleep in monotonous situations and may not be aware of sleep intrusions during the day. Caregivers’ reports are therefore essential for a timely identification [126].

The SCOPA-S includes questions about daytime sleepiness during the past 4 weeks [136, 138]. The Epworth Sleepiness Scale (ESS) was purposely designed to capture and grade daytime sleepiness [99], with a cutoff > 10 points marking excessive daytime sleepiness, and is applicable in PD settings. Many affected patients may however lack an awareness for the problem, with substantial discrepancies between subjectively perceived and objectively measured daytime sleepiness [13].

**Table:** 

Recommendation	
Besides patient history and caregiver reports, the ESS is a validated and recommended screening tool for daytime sleepiness in PD	
Level of consensus: 100%, strong consensus	

#### How to diagnose excessive daytime sleepiness in PD?

The Multiple Sleep Latency Test (MSLT) and the Maintenance of Wakefulness Test (MWT) are considered the gold standard for objectively evaluating daytime sleepiness. Both tests require a sleep laboratory setting. The MWT measures the ability to stay awake in a monotonous situation [147], whereas the MLST evaluates the time it takes to fall asleep in a monotonous situation [9, 34]. Due to these opposing concepts, the concordance between both tests is low. Of note, neither the MSLT nor the MWT have been validated in PD. Assessments in individuals with obstructive sleep apnea (OSA) also showed discrepancies between ESS scorings and objective measurements [18, 32, 72]. MWT and driving simulation tests in OSA patients correlated with regard to driving capability [174]. However, there is no evidence that MWT results correspond to driving accidents in the real world [174]. ESS and MSLT did not, but TAP-test and Psychomotor Vigilance Test Reaction Time results did correlate to driving simulator performance in OSA patients [95]. In PD, studies show that the subjective perception of sleepiness and the objectively measured probability of falling asleep unintentionally may deviate [13]: every fourth PD individual did not perceive any sleepiness prior to daytime sleep tests, which objectively demonstrated sleep episodes. Evaluation of driving capability or potentially accident-prone daytime sleepiness in the work environment should therefore not rely on subjective perception, although there is no objective gold standard for diagnosing daytime sleepiness in PD individuals at present. VPSG-based assessment of nocturnal sleep quality is an important prerequisite for interpreting daytime vigilance tests properly and identifying potential causes of daytime sleepiness. Several studies demonstrated however an insufficient correlation between nocturnal sleep quality and subjective or objective measurements of daytime sleepiness [123, 217]. Self-perceived daytime sleepiness was also not consistently reflected in MSLT [217]. Abovementioned observations altogether indicate that the mechanisms underlying daytime sleepiness in PD are currently poorly understood and that tools for detecting and diagnosing daytime sleepiness in PD are insufficient.

**Table:** 

Recommendation	
For a diagnosis of daytime sleepiness, a detailed medical history, caregiver reports, questionnaires such as ESS, as well as an assessment of possible causes of daytime sleepiness are recommended. An assessment of nocturnal sleep quality by means of vPSG, as well as MSLT and other neuropsychological test procedures including PVT or driving simulator test may be considered	
Level of consensus: 92.6%, consensus	

#### How to treat excessive daytime sleepiness in PD?

If the diagnostic workup identifies a sleep disorder as a possible cause for daytime sleepiness, an etiology-driven therapeutic approach according to the guidelines summarized above is recommended. Especially motor or non-motor wearing-off phenomena inducing nocturnal sleeplessness call for the adjustment of dopaminergic medications. Total levodopa-equivalent dose should be critically appraised and the possibility of a dose-reduction or medication redistribution should be considered, since suboptimal medication timing may contribute to sleeplessness and impaired daytime vigilance [70]. Sleep attacks associated with dopamine agonists [78] need to be excluded. A switch to amantadine [207] or invasive therapies may prove beneficial.

Non-pharmacological approaches include improvement of sleep hygiene with regular bedtimes and naps, social activation, bright light application to regulate circadian rhythm and intensified physical activities [244]. Intensive sport improved subjective sleep quality [244] and power training has been shown to improve both sleep and cognition [258].

If all treatable causes of excessive daytime sleepiness have been excluded and non-pharmacological measures prove insufficient, medication may be considered, although evidence is poor. Up to 400 mg of caffeine showed minimal, clinically non-meaningful effects on the ESS [175]. A meta-analysis of studies investigating modafinil in PD, using dosages of 200–400 mg as per prescribing information, demonstrated a reduction of subjective, but not objectively measured daytime sleepiness [188]. An open-label study showed preliminary positive effects for methylphenhidate [51]. Istradefylline, a selective adenosine A2A receptor antagonist, improved daytime sleepiness in an open label study [221], but is not licensed in Europe. Gamma hydroxybutyric acid improved PSG-based measurements in a double-blind randomized phase II study, but had a severely unfavorable safety profile and cannot be therefore recommended [51]. The selective noradrenaline-reuptake inhibitor atomoxetine improved daytime sleepiness and cognition in a pilot randomized double-blind study in PD individuals [253]. Medication for comorbid diseases and other medications, especially hypnotics and sedating antidepressants should be reviewed critically. Ultimately, the MDS recommends to prohibit driving as long as daytime sleepiness persist [70].

**Table:** 

Recommendation	
For treatment of daytime sleepiness in PD, a cause-oriented diagnostic work-up is recommended Daytime sleepiness due to motor and non-motor complications of PD should be treated by optimizing PD therapy Non-pharmacological therapies such as sleep hygiene, exercise and light therapy should be recommended Primary sleep disorders responsible for daytime sleepiness should be diagnosed and treated accordingly. Only in case of persistent daytime sleepiness, pharmacological options (i.e., modafinil) may be considered on an individual basis, though the available evidence is low	
Level of consensus: 100%, strong consensus	

#### What are the effects of nocturnal sleep disturbances on PD individuals and their relatives?

Numerous cross-sectional cohort studies show that insomnia and sleep disorders are associated with a reduced quality of life and an increased disease burden in PD individuals [105, 118, 243, 264]. An association between sleep disturbances and depression has been found in PD individuals and their partners who may also develop sleep disturbances [213, 248]. Even mild cases of comorbid SDB are associated with cognitive impairment [178] and motor performance deteriorates in case of comorbid sleep disorders [159, 199].

**Table:** 

Recommendation	
Sleep disturbances have a pronounced negative impact on PD individuals and their partners, especially regarding motor performance, quality of life, mood and the nocturnal sleep quality of partners. Sleep disorders should be therefore routinely considered in the clinical diagnostic work-up and therapeutic plan Therapeutic decisions should not only consider the concerns of PD individuals, but also consequences for their partners and caregivers	
Level of consensus: 96,8%, strong consensus	

#### What are the effects of daytime sleepiness on PD individuals and their relatives?

Increased daytime sleepiness and unintentionally falling asleep impair the ability to drive and thus restrict the individual mobility. Sleep attacks in daily life situations represent a hazard and have a negative impact on quality of life [6, 38, 214, 262]. A longitudinal cohort study showed that daytime sleepiness increases as the disease progresses [6]. Although no explicit studies could be found, it is evident that unintended falling asleep, impaired attention and participation due to increased daytime sleepiness inhibit communication and social engangement, implying a bidirectional influence.

**Table:** 

Recommendation	
Patients with increased daytime sleepiness are not capable of driving; in addition they suffer impairment in many aspects of communication and social participation This impacts negatively on their social environment	
Level of consensus: 92.6%, consensus	

#### What is the prognosis of PD individuals with sleep disorders as compared to those without and which sleep disorders impact most?

Apart from the pathophysiological connection between sleep and neurodegeneration, clinical evidence shows that sleep disorders are associated with a faster disease progression [25, 202, 220, 266]. Especially RBD is associated with a more severe PD course of the disease, earlier motor complications and cognitive decline, indicating that RBD represents a progression marker in PD [157, 176]. Changes in EEG microstructure during sleep, such as a reduction of delta waves and spindles, are specifically associated with a more rapid cognitive decline [52]. In the general population, SDB is associated with metabolic complications and cardiovascular disease. In PD individuals, SDB additionally impact on executive function, working memory and motor performance [56].

**Table:** 

Recommendation	
Sleep disorders are associated with more rapid disease progression RBD in early stages of PD predicts a more severe disease course with earlier cognitive, autonomic and motor complications	
Level of consensus: 96.2%, strong consensus	

## Discussion

Identifying and treating autonomic failure with a stepwise combination of non-pharmacological and pharmacological options is essential for improving the symptomatic burden of PD individuals and reducing the risk of life-threatening complications such as recurrent urinary tract infections, syncope-related falls, hypertensive emergencies, gastrointestinal occlusions and perforations. The type and time-course of autonomic failure may also contribute to the differential diagnosis of PD from atypical Parkinsonian syndromes like multiple system atrophy, in which an earlier, and more severe autonomic impairment involving multiple domains is frequently observed [60, 65].

For an effective, etiology-driven pain treatment in PD individuals, it is essential to distinguish between nociceptive, neuropathic and nociplastic pain types by means of detailed history taking and PD-specific scales and questionnaires. It is to be born in mind that multiple pain causes may be present in single PD individuals and that a bidirectional interplay often exists between painful sensations and PD motor symptoms, especially in fluctuating patients. Pain treatment should therefore begin with an adjustment of dopaminergic medications whenever pain is reckoned to depend upon insufficient dopaminergic stimulation and progressively integrate pharmacological measures targeting any identified cause of nociceptive or neuropathic pain, until an adequate symptomatic control is achieved.

**Fig. 2:**
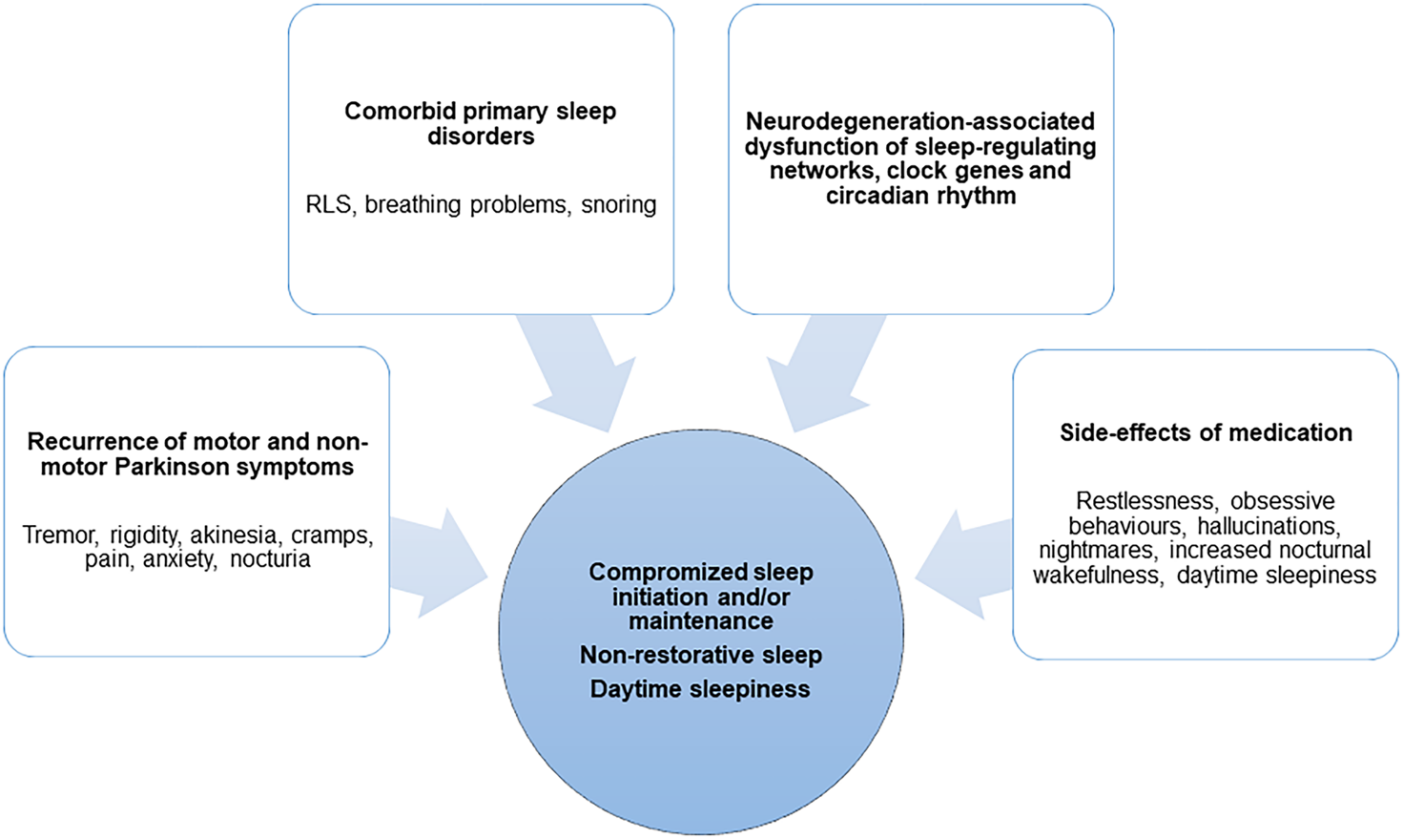
Symptoms and causes of sleep disturbances in PD. *RLS* restless legs syndrome

**Fig. 3:**
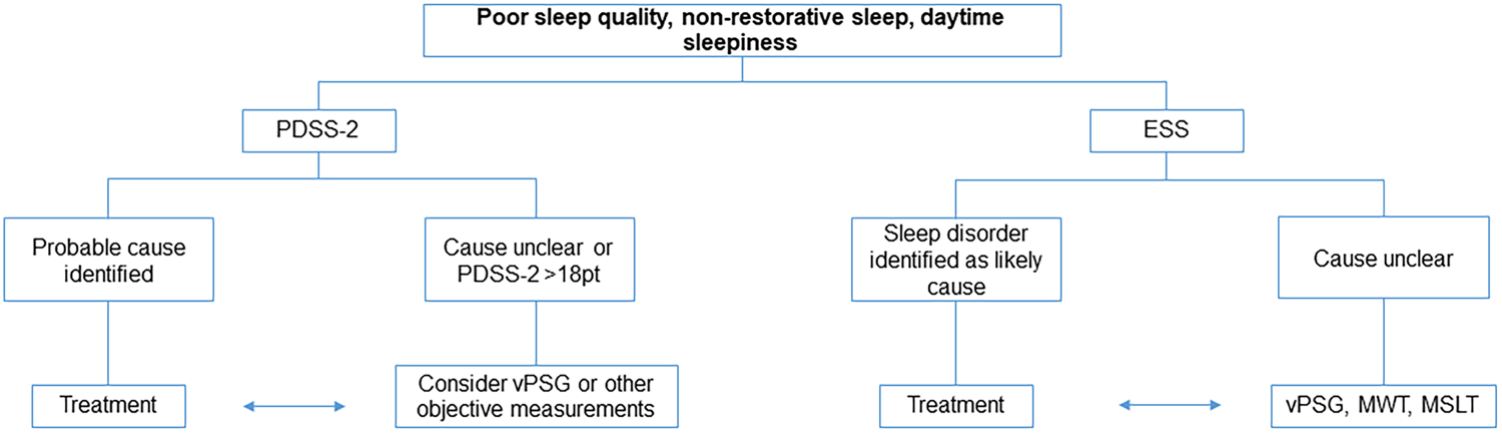
Diagnostic algorithm for sleep problems and excessive daytime sleepiness in PD. *ESS* Epworth Sleepiness Scale, *MSLT* Multiple Sleep Latency Test, *MWT* Maintenance of Wakefulness Test, *PDSS-2* Parkinson’s Disease Sleep Scale-2, *vPSG* video-supported polysomnography

The pathophysiology of sleep regulation in PD individuals with its spectrum of clinical manifestations needs to be contextualized in the neurodegenerative process of the disease to deduce diagnostic procedures and appropriate therapeutic steps (Fig. 2). To this end, the interdependence of disturbed nocturnal sleep and diurnal wakefulness with quality of life, disease burden, mood, motor performance, cognition, social interaction, disease progression as well as caregiver burden should be considered. An algorithm for the diagnostic work-up of sleep disturbances and daytime sleepiness in PD is proposed in Fig. 3. The identification of the underlying sleep disorder drives the specific therapeutic approach, which is based on PD-specific measures and also refers to guidelines for other stand-alone disease entities such as RLS, insomnia or SDB.

In conclusion, acknowledging the time- and resource constrains [86] frequently encountered by physicians in everyday practice, especially after COVID-19 pandemic outbreak [64, 182], yet aiming at maximizing the quality of healthcare provision to individuals living with PD, the 2023 edition of the PD guidelines of the German Society of Neurology provides evidence- and consensus-based recommendations for a practice-oriented and etiology-driven stepwise approach to the diagnosis and treatment of motor and non-motor PD symptoms, including autonomic failure, pain and sleep disturbances. Future trials should ideally close the gaps in evidence for the pharmacological and non-pharmacological treatment of these, still underestimated, disturbances in the specific setting of PD.

## Data Availability

The authors confirm that the data supporting the present recommendations are summarized in the body of manuscript and the referenced literature.
